# Using PCR-Based Detection and Genotyping to Trace *Streptococcus salivarius* Meningitis Outbreak Strain to Oral Flora of Radiology Physician Assistant

**DOI:** 10.1371/journal.pone.0032169

**Published:** 2012-02-22

**Authors:** Velusamy Srinivasan, Robert E. Gertz Jr., Patricia L. Shewmaker, Sarah Patrick, Amit S. Chitnis, Heather O'Connell, Isaac Benowitz, Priti Patel, Alice Y. Guh, Judith Noble-Wang, George Turabelidze, Bernard Beall

**Affiliations:** 1 Respiratory Diseases Branch, Division of Bacterial Diseases, Centers for Disease Control and Prevention, Atlanta, Georgia, United States of America; 2 Section of Epidemiology for Public Health Practice, Division of Community and Public Health, Missouri Department of Health and Senior Services, St. Louis, Missouri, United States of America; 3 Surveillance Branch, Division of Healthcare Quality Promotion, Centers for Disease Control and Prevention, Atlanta, Georgia, United States of America; 4 Epidemic Intelligence Service, Office of Workforce and Career Development, Centers for Disease Control and Prevention, Atlanta, Georgia, United States of America; Cairo University, Egypt

## Abstract

We recently investigated three cases of bacterial meningitis that were reported from a midwestern radiology clinic where facemasks were not worn during spinal injection of contrast agent during myelography procedures. Using pulsed field gel electrophoresis we linked a case strain of *S. salivarius* to an oral specimen of a radiology physician assistant (RPA). We also used a real-time PCR assay to detect *S. salivarius* DNA within a culture-negative cerebrospinal fluid (CSF) specimen. Here we extend this investigation through using a nested PCR/sequencing strategy to link the culture-negative CSF specimen to the case strain. We also provide validation of the real-time PCR assay used, demonstrating that it is not solely specific for *Streptococcus salivarius*, but is also highly sensitive for detection of the closely related oral species *Streptococcus vestibularis*. Through using multilocus sequence typing and 16S rDNA sequencing we further strengthen the link between the CSF case isolate and the RPA carriage isolate. We also demonstrate that the newly characterized strains from this study are distinct from previously characterized *S. salivarius* strains associated with carriage and meningitis.

## Introduction

*Streptococcus salivarius* is a predominant bacterial species of human oral flora [1], [2], and has been the most commonly identified species causing bacterial meningitis cases that occur after spinal injection procedures because of contamination of the procedure site with saliva [3]. The reliable density of this species within human saliva allows for its use in forensic detection using DNA amplification [2]. Analysis based upon sequences of multiple chromosomal loci indicates that *S. salivarius* is a distinct, genetically diverse species that undergoes frequent intraspecies horizontal exchange events [4], [5], [6]. A highly related species, *Streptococcus vestibularis*, is also found as common oral flora. There is cumulative sequence-based data that reveal the likelihood of frequent genetic exchange between the two species but also supports their separation into distinct species [5].

Independent cases of *S. salivarius* meningitis epidemiologically linked to the same spinal injection procedure provider have previously been reported [6], [7], [8]. In two situations genetically indistinguishable strains were recovered from the case CSF specimen and the oral flora of the individual carrying out the lumbar puncture [6], [8].

More recently (11/8/2010) the CDC *Streptococcus* Lab received two isolates from the CSF of a case patient that was culture-positive for alpha-hemolytic streptococci and also received a small amount (80 µL) of refrigerated culture-negative CSF from a second case patient. Neither isolates nor CSF specimens were available from the third case patient who was treated presumptively for bacterial meningitis without specimen collection. All three patients had received myelograms from the same radiology physician assistant (RPA) and technician on the same day (10/25/2010). In addition, on 11/8/2010 the CDC *Streptococcus* Lab received saliva and dorsal tongue specimens collected by a local public health nurse as part of the investigation from the RPA and the technician transported in Amies medium. This outbreak of three cases of bacterial meningitis at an outpatient radiology clinic was previously described in detail [9]. The *S. salivarius* CSF isolate was indistinguishable from the oral *S. salivarius* strain carried by the RPA using pulsed field electrophoresis but was distinct from the *S. salivarius* strain carried by the technician. CSF from a culture-negative case was found to be positive using a real-time PCR assay reportedly specific for *S. salivarius* [10]. In the following sections we describe concurrent and subsequent lab testing not described in the previous epidemiologic account [9]. This testing includes our validation of the real-time *S. salivarius* PCR assay, multilocus sequence/16 s rDNA- based speciation and resolution of case and carriage isolates, 16 s rDNA, and details of culture-based isolation and speciation of isolates. We also describe the usage of nested PCR and DNA sequencing to identify a specific signature within this culture-negative CSF that was shared by the case strain.

## Materials and Methods

### Isolation and species identification

Saliva and dorsal tongue specimens were initially subjected to broth-enrichment in Todd-Hewitt broth as previously described [6] prior to plating serial dilutions upon Trypticase Soy Agar with 5% sheep blood (BAP), colistin nalidixic acid agar (CNA), and Mitis-salivarius agar with 1% potassium tellurite (MSA) [Becton Dickinson], which is highly effective for isolation of the *Streptococcus mitis* group, *S. salivarius*, and enterococci from heavily contaminated specimens [11]. Species identification of the meningitis case strain and carriage isolates used a conventional biochemical identification scheme [12], and the rapid ID32 STREP method [13] as described by the manufacturer (bioMérieux, Inc, Durham, NC). Species identification at the genetic level was confirmed using the MLST-based system described at http://viridans.emlsa.net/ [4].

### DNA extraction from culture-negative CSF

DNA was extracted from the 80 µL culture-negative CSF specimen using a modified method of Qiagen Blood/Tissue DNA extraction kit (Qiagen Inc., Valencia, CA; Table S1; this and other Streptococcal laboratory methods methods are available at http://www.cdc.gov/ncidod/biotech/strep/protocols.htm) and eluted in 30 µL of TE buffer. From this, five µL of DNA was used in the real-time PCR reaction.

### *S. salivarius* real-time PCR assay validation

Sixteen strains of *S. salivarius* from clinical origin, six type strains of species belonging to the *S. salivarius* group (includes *S. salivarius*, *S. vestibularis*, *S. thermophilus*), 57 strains belonging to 31 additional streptococcal species, as well as 1–4 strains of nine non-streptococcal species were used to validate specificity of the primers and probe. *Streptococcus salivarius* ATCC 7073 was used as a positive control in all assays. The primer and probe sequences were GtfP-F (5′-CAC GCC ATG CTG GAA GTG3′), GtfP-R (5′GCG ATG AGC CAA GCT GAA G3′) and GtfP-probe (5′-FAM-TTA GCT GCT GCG TAG ACT TCG TCT-BHQ1-3′) as previously described [10]. The concentrations for each primer (100, 200, 300, 400 and 500 nM) and probe (100, 200 and 300 nM) were optimized in individual assays and optimal concentrations were used in validation assays. All real-time PCR reactions were carried out in 25 µl reaction volumes by using the QuantiTect multiplex PCR NoRox Mix (Qiagen), with five µl of sample DNA. The optimized primers (200 nM each) and probe (100 nM) concentrations were used for further experiments. Lower limits of detection were assessed with 10-fold serial dilutions of purified DNA of *S. salivarius* ATCC 7073. The PCR reactions were carried out in Stratagene Mx3000P system (Stratagene, La Jolla, CA) with the conditions described previously [10] and amplification data were analyzed using the Mx3000P software. DNA was extracted from the isolates by Chelex 100® (Bio-Rad Laboratories, Hercules, CA) kit according to the manufacturer's instructions.

### Multilocus sequence typing (MLST), 16S rDNA sequencing, and nested PCR

MLST and 16S rDNA amplification/sequencing of *S. salivarius* strains was carried out as previously described [4], [5], [6]. For partial genotyping of *S. salivarius* DNA extracted from culture-negative CSF specimen, we used the previously described ddlA-up and ddlA-dn primers [5] for initial amplification, followed by a nested PCR step. The expected 531 bp PCR fragment was purified from a 1% agarose gel using Qiagen Gel purification kit (Qiagen). The purified PCR product was used as template and a nested 475 bp PCR fragment was generated employing primers: ddlAseq1 (5′-TTC TTG AAG TTG CTG GTG TGC CTC-3′) and ddlAseq2 (5′-GTC AGG AAG AAA TCA CAG CGG GC-3′). The nested PCR fragment was directly purified and sequenced using primers ddlAseq1 and ddlAseq2.

### GenBank accession numbers for new sequences

Nine new alleles are paired here with their provided accession numbers, including *ddlA31*/JN819659, *thrS22*/JN819660, *pyrE26*/JN819661, *dnaE25*JN819662, *rpoB2*/JN819663, *pyk1*/JN819664, *ppaC2*/JN819665, *pfl1*/JN819666, and *map1*/JN819667.

## Results

### Species identification of meningitis case CSF isolate and isolation of *S. salivarius* from oral specimens of clinic staff

All broth-enrichment cultures of the saliva and dorsal tongue specimens taken from the RPA and attending technician yielded normal flora on non-selective BAP plates and typical Gram-positive bacterial colonies upon CNA plates. The characteristic large mucoid, domed morphology of *S. salivarius* was the predominant colony morphology observed on MSA plates. These isolates and the two CSF isolates were identified as *S. salivarius* using the Rapid ID32 Strep method (99.9% confidence level) and using the conventional biochemical scheme [12]. Interestingly, both the case *S. salivarius* isolates and multiple carriage *S. salivarius* isolates from the RPA gave negative or weakly positive reactions for trehalose with both biochemical testing methods while *S. salivarius* isolates from the technician were strongly trehalase-positive. All strains were unambiguously identified as *S. salivarius* using the sequence based approach at http://viridans.emlsa.net/ [4] that clusters species based upon comparisons of a 3063 bp DNA sequence that consists of 7 concatenated housekeeping gene fragments (*rpoB*, *sodA*, *pyk*, *ppaC*, *tuf*, *pfl*, and *map*) shown in Table 1.

**Table 1:** Multilocus allele and 16S rRNA gene sequence designation depicting comparison of *S. salivarius* strain from meningitis patient to oral carriage isolates recovered from radiology physician assistant and technician.^a^

Isolates	*glcK*	*ddlA*	*pepO*	*ilvC*	*thrS*	*pyrE*	*dnaE*	*sodA*	*rpoB*	*sodA2*	*pyk*	*ppaC*	*tuf*	*pfl*	*map*	16S *rrnA*
CSF^b^	7	***31***	3	4	***22***	***26***	***25***	14	***2***	1	***1***	1	1	***1***	***1***	2
RPAcarriage^c^	7	***31***	3	4	***22***	***26***	***25***	14	***2***	1	***1***	1	1	***1***	***1***	2
Technician^d^	4	3	3	4	14	12	17	14	1	1	2	***2***	2	***1***	***1***	1

a The 8 housekeeping gene targets *glcK*, *ddlA*, *pepO*, *ilvC*, *thrS*, *pyrE*, *dnaE*, and *sodA* refer to the *S. salivarius*group MLST scheme described in reference 5 and the alleles correspond to the same ones listed in reference 5 except where in bold and italicized. The 7 remaining housekeeping gene targets (*rpoB*, *sodA2*, *pyk*, *ppaC*, *tuf*, *pfl*, *map*) refer to the viridans streptococcal MLST/speciating scheme available at http://viridans.emlsa.net/ (5). All bold/italicized designations correspond to sequences not found in the GenBank that were found in these isolates. GenBank accessions for the sequences corresponding to all of the underlined numbers are supplied in the Methods. The 8 following *S. salivarius* sequences have been previously documented by other investigators and are listed along with their GenBank accession numbers: *rpoB1*/GU556187, *sodA2-1*/AB200166, *ppaC1*/GU556189, *pyk2*/CP002888, *tuf1*/GU556190, *tuf2*/CP002888, 16S *rrnA2*/CP002888, and 16S *rrnA1*/GU175444.

b Results shared between two isolates from case CSF.

c Results shared between three carriage isolates (dorsal tongue and saliva).

d Results shared between two carriage isolates (dorsal tongue and saliva).

### Comparisons of MLST profiles implicate carriage *S. salivarius* strain recovered from RPA as etiologic agent

We utilized DNA sequence based typing of 16 targets (Table 1) to compare the meningitis case strain to saliva and dorsal tongue *S. salivarius* isolates recovered from the RPA that performed the myelograms as well as from the attendant technician. The two CSF case isolates from the case patient and all 3 tested isolates from the RPA were genetically indistinguishable using MLST and 16 s rDNA sequence analysis (Table 1). In contrast, oral *S. salivarius* isolated from the technician who was also in the procedure room during the myelography procedures shared only six of 16 identical loci (Table 1). Neither profile showed high similarity to strains previously genotyped using this approach [4], [5], [6]. Comparison of the meningitis case/RPA carriage strain to another recently characterized *S. salivarius* strain associated with meningitis [6] also revealed only six of 16 identical loci (data not shown). The carriage strain recovered from the technician associated with this investigation showed slightly more relatedness to this recently characterized meningitis strain with eight of 16 identical loci (data not shown).

### Detection of *S. salivarius* or *S. vestibularis* DNA within culture-negative CSF of second meningitis patient

A previously described real-time PCR assay for *S. salivarius* [10] was validated using an extensive panel of bacterial strains, including 37 streptococcal and nine non-streptococcal species (not shown). The primers and probe were highly specific for both *S. salivarius* and the closely related species within the *S. salivarius* group, *Streptococcus vestibularis*. We found that for both of these species the assay had a detection limit of <10 copies/assay. The assay was negative for all other species, including the other recognized *S. salivarius* group member, *S. thermophilus*, as well as three additional species that have been assigned within some taxonomic schemes to the *S. salivarius* group (*S. infantarius*, *S. alactolyticus*, and *S. hyointestinalis*) [12]. The analytical lower detection limit for the real-time assay was measured by amplifying serial dilutions of purified DNA from positive control strain *S. salivarius* ATCC 7073. The standard curve generated had slope of −3.3, with the *R* ^2^ value being >0.99 and the efficiency was 100%.

The real-time PCR assay of the DNA extract prepared from the culture-negative CSF specimen revealed a repeatable, but relatively high C*_T_* value of 37 that we nonetheless interpreted as positive for *S. salivarius* and/or *S. vestibularis*. We further confirmed the presence of *S. salivarius* in the CSF sample by repeating the real time PCR in triplicate. We tentatively attributed the low signal to the small volume of CSF (80 µL), use of antibiotics prior to CSF collection, and possible degradation of *S. salivarius* DNA due to long (>13 days) storage of the unfrozen specimen.

### Detection of case strain sequence signature within culture-negative CSF

Employing a nested PCR and sequencing strategy described in the Methods, we found that the 428 bp fragment remaining after subtraction of the 47 bp contributed by primer sequences shared identity with *ddlA31* over its entire overlap. The *ddlA31* sequence is unique to the *S. salivarius* case strain and the three carriage isolates obtained from the RPA. The closest known match to this 428 bp sequence is *ddlA12* [5], which shares 97.9% identity (nine nucleotide differences).

## Discussion

Alpha-hemolytic streptococcal meningitis has rarely been reported in connection with the performance of lumbar puncture for various purposes including myelograms and spinal anesthesia [3], but since September of 2008, we have become aware of eight meningitis cases of this nature due to *S. salivarius*. Besides the most recent three cases in Missouri [9] that are the subject of our report, these include three New York cases that were associated with one anesthesiologist [6] and two Ohio cases that were associated with another anesthesiologist [14]. For both the prior New York outbreak associated with an anesthesiologist [6] and our further investigation of the outbreak involving the RPA [9] presented here, compelling evidence on the route of transmission was supplied based upon unique 16 locus profiles shared solely between oral *S. salivarius* isolates of the administering anesthesiologist or RPA and the patient receiving the spinal injection procedure. Also, for the study presented here we were able to provide more compelling evidence linking the RPA oral *S. salivarius* strain to the patients' infections by demonstrating a unique *ddlA* signature shared with the *S. salivarius* DNA detected in a culture-negative CSF from a second meningitis case patient.

According to one review [3], 55 cases of spinal injection-related bacterial meningitis occurred during 1952 to 1998, and all were caused by streptococcal species. Although usually of unidentified alpha-hemolytic species, *S. salivarius* was the most common species identified (19 cases, eight of which involved myelography). Since alpha-hemolytic streptococci are often not identified at the species level [12], it is possible that even more of these meningitis cases were due to *S. salivarius*. A very recent review of *S. salivarius* meningitis [15] includes information regarding 23 additional cases of *S. salivarius* meningitis associated with spinal injection procedures that have occurred during the period 1999–2009. There have been several instances of clustering of streptococcal meningitis cases associated with such procedures [7], [9], [14], [16]–[Bibr pone.0032169-Gelfand1] [18]. This observed clustering, the known high density of oral streptococci (especially that of *S. salivarius*), and instances of apparent genetic identity shared between the carriage isolates of the operator and the etiologic agent cumulatively demonstrate that the individual performing spinal injection procedures is the source of the etiologic agent in these cases of *S. salivarius* meningitis. It should now be very clear that strict adherence to aseptic technique is essential, especially with regard to usage of face masks in preventing transmission of saliva into the central nervous system upon performing procedures involving spinal injection [19], [20]. The rapidity of *S. salivarius* growth upon entry into the spinal column is evident from the typically rapid onset of meningitis (within 7–24 h) [3], [9], [14], [21].

We have found that the MLST-based method of Bishop and colleagues [4], using their degenerate amplification and sequencing primers, is a very useful method for nonsubjective speciation of diverse viridans streptococci using comparisons of concatenated housekeeping gene allele sequences which is also useful for strain typing using the allele combinations. In our hands we find that MLST genotyping is easier to perform and relatively cheaper than PFGE. Even when dealing with strains within the same streptococcal species we find a broad range of difficulty in generating distinct PFGE profiles. Using this molecular speciation/strain genotyping methodology (see http://viridans.emlsa.net/) for a recent investigation involving oral contamination, we found it very straightforward to identify five distinct viridans streptococcal species and multiple distinct clones within each species (unpublished data). As in the situation described in this report, we typically found multiple alleles shared between unrelated strains (strains sharing less than four of the 7–8 housekeeping gene sequence markers described in either of the two MLST schemes [4], [5]). Nonetheless, when confronted with an investigation where a lab is attempting to link any of a large number of carriage or case isolates to specific outbreak strains, it is often useful to limit the allelic comparison of the initial isolate set to only one or two loci in order to rapidly remove all or most unrelated isolates from the analysis. Although we used two different MLST systems [4], [5] for the work described here, we have found that for outbreak investigations the 7–8 independent loci provided by either system suffices for resolving *S. salivarius* strains. It should be mentioned, however, that the degenerate primer approach [4] is most relevant for targeting unknown viridans streptococci for rapid speciation as well as genotyping.

The MLST method of assessing clonal relatedness between viridans streptococci is a very sensitive and non-subjective typing method, especially in light of the high degree of recombination apparent in these species [4], [5]. With this in mind, it would be an advance for research and outbreak investigations of these diverse streptococcal species if this MLST system could adapt MLST allele and type designations modeled after the www.mlst.net internet site. For example, prevalent strains of *Streptococcus pyogenes*, *Streptococcus agalactiae*, and *Streptococcus pneumoniae* recovered from clinical sources or carriage specimens are readily recognizable globally through MLST designations provided at this internet site. Equivalent knowledge of strains of the various viridans species that might be predominant in carriage or potentially preferentially associated with disease manifestations is entirely lacking.

## Supporting Information

Table S1 Protocol for blood and body fluid DNA extraction optimized for streptococcal DNA.
(PDF)
