# Supplementary material for: Using PCR-Based Detection and Genotyping to Trace Streptococcus salivarius Meningitis Outbreak Strain to Oral Flora of Radiology Physician Assistant
Source: PLoS One. 2012 Feb 22;7(2):e32169. doi: 10.1371/journal.pone.0032169 (PMC3285201; doi:10.1371/journal.pone.0032169)
Supplement: Table S1 — Protocol for blood and body fluid DNA extraction optimized for streptococcal DNA. (PDF) [file pone.0032169.s001.pdf]

Blood and Body Fluid DNA Extraction for Streptococci

- When using a new QIAampDNA Mini kit or DNeasyBlood & Tissue Kit, add alcohol to buffers AW1 and AW2 as indicated on the bottles.
- Equilibrate samples and AE Buffer to room temperature (15-25 ^{o}C).
- Heat the water bath to 56 ^{o}C.
- If a precipitate has formed in Buffer AL, dissolve by incubating at 56 ^{o}C.
- All centrifugations steps should be carried out at room temperature.

- Pipet 100 uL of TE buffer containing 0.04g/mL of lysozyme + 75 U/mL of mutanolysin (freshly prepared) into the bottom of a 1.5 mL microcentrifuge tube.
    - **TE buffer (10mM Tris-HCl, 1 mM EDTA, pH 8.0)**
    - **The lysozyme (Sigma-L-6876), and mutanolysin (Sigma-M9901) should be added to the TE buffer right before the use (freshly prepared).**
- Add 200 uL of the sample. Vortex and incubate at 37 ^{o}C in water bath for 1 hour.
- Add 20 uL of proteinase K (600 mAU/ml or 40 mAU/mg – Qiagen cat. # 19133). Vortex tube briefly.
- Incubate at 56 ^{o}C in water bath for 30 min.
- Centrifuge briefly to remove drops from inside of the lid.
- Add 200 uL of Buffer AL. Vortex tube briefly.
- Incubate at room temperature for 10 min.
- Add **260 uL** ethanol (96-100%) to the sample, and mix again by pulse-vortexing for 15s. After mix, briefly centrifuge the tube to remove drops from the inside of the lid.
- Pipet the mixture into the QIAamp Spin column sitting in the 2 mL collection tube without wetting the rim, close the cap.

- Centrifuge at 6000 x *g* or 8000 rpm for 1 min. Discard eluate and collection tube.
- Place mini column into fresh collection tube. Add 500 uL of Buffer AW1 without wetting the rim, close the cap and centrifuge 1 min. at 6000 x *g* or 8000 rpm. Discard eluate and collection tube.
- Place mini column into fresh collection tube. Add 500 uL Buffer AW2 and centrifuge for 3 minutes at FULL SPEED (20,000 x *g* or 14,000 rpm) to dry column. Discard eluate and collection tube.
- Place mini column in 1.5 ml sterile eppendorf tube. Add 100 uL Buffer AE onto membrane. Incubate room temperature 5 minutes. Centrifuge 1 min. at 6000 x *g* or 8000 rpm to eluate DNA. (Keep at -20 ^{o}C until use).
